# Eighteen cases of renal aneurysms: Clinical retrospective analysis and experience of endovascular interventional treatment

**DOI:** 10.3389/fsurg.2023.1106682

**Published:** 2023-02-28

**Authors:** Tao Lu, Bin Lin, Yan-ping Zhang, Jian-hui Zhang, Jie-Wei Luo, Yi Tang, Zhu-Ting Fang

**Affiliations:** ^1^ Fujian Provincial Hospital, Shengli Clinical Medical College of Fujian Medical University, Fuzhou, China; ^2^ Department of Interventional Radiology, Fujian Provincial Hospital, Fuzhou, China

**Keywords:** renal artery aneurysms, retrospective analysis, endovascular treatment, safety and efficacy, clinical features

## Abstract

**Background:**

Development of endovascular interventional techniques gradually replaced traditional open surgery and has become the preferred treatment for renal aneurysms. This study aimed to analyze the clinical characteristics of renal artery aneurysm (RAA) and the safety and efficacy of intravascular interventional treatment.

**Materials and Methods:**

We retrospectively analyzed the clinical characteristics and imaging data of 23 aneurysms in 18 patients with RAA. The technical success rate, complication rate, mortality rate, reintervention rate, and use of embolization materials were evaluated.

**Results:**

In 18 patients with RAA (age, 32–72 years, average age, 52.2 ± 11.2 years), a total of 23 aneurysms were found (diameter 0.5–5.5 cm, average diameter 2.2 ± 1.4 cm). Among them, 11 cases (61.1%) were discovered accidentally, and the remaining patients were diagnosed due to the following major complaints: four cases (22.2%) presented low back pain, two (11.1%) were due to high blood pressure, and one (5.5%) had low back pain with gross hematuria. A total of 14 aneurysms in 13 patients received endovascular interventional therapy. The technical success rate of 13 patients with renal aneurysms was 100%. Three of the 18 patients were lost to follow-up, and the remaining were followed up for 4–89 months. There was no recurrence of the aneurysm or displacement of the stent or coil.

**Conclusion:**

Endovascular treatment for RAA has a high success rate, low complication rate, and low reintervention rate. It has the advantage of less trauma and is flexible and more targeted for different types of renal aneurysms.

## Introduction

Aneurysm-like lesions of renal blood vessels are abnormal expansions of the vascular lumen, which can be divided into true aneurysms and pseudoaneurysms according to the integrity of the vessel wall. True aneurysms retain all three layers of the vascular wall (intima, media, and adventitia), while pseudoaneurysms cause the continuity of the artery wall to be interrupted by various factors and blood spills into the surrounding tissues to form a hematoma (1). This study describes the clinical characteristics and treatment methods for true renal aneurysms.

Renal artery aneurysms (RAAs) are rare, accounting for 1% of all aneurysms and 15%–22% of visceral aneurysms (2). The incidence of RAA in the general population and angiography and computed tomography (CT) research reports were 0.1% and 0.3%–2.5%, respectively. The incidence of RAA rupture is 3%–5% and the mortality rate after non-pregnant aneurysm rupture is approximately 10% (3, 4). Massive hemorrhage caused by RAA rupture is a clinical emergency that requires urgent intervention. Therefore, it is necessary to identify patients with RAA with high-risk rupture risk factors early and implement appropriate interventions to treat ruptured aneurysms.

With the development of vascular and interventional radiology, interventional therapy has become less invasive, with fewer complications, and has become the first choice for the clinical treatment of RAA. This study included 18 patients with true renal aneurysms. This study aimed to retrospectively analyze clinical characteristics and imaging data, evaluate the safety and efficacy of endovascular interventional therapy, and share the clinical experience with the readers.

## Methods and materials

### Clinical information

We searched for data on aneurysms using the keyword “renal artery aneurysms” in the imaging radiology system from July 2014 to mid-June 2021. According to pathogenesis, clinical, and morphological criteria, a total of 18 patients with true renal aneurysms, including 23 aneurysms, were enrolled after excluding pseudo-renal aneurysms. I In order to ensure more complete and rich data, the selected patients with aneurysms were reviewed and not eliminated again: the included patients with renal aneurysms included patients with indications for treatment after evaluation by experts. If the complex renal aneurysms were not suitable for endovascular interventional treatment, they should be referred to surgical treatment. According to medical records, sex, age, comorbidities, clinical manifestations, number and location of aneurysms, treatment measures and materials, and prognosis of the patients were recorded. We recorded the incidence of complications, mortality, and reintervention rate after endovascular treatment.

### The treatment process of RAA

**Figure 1:**
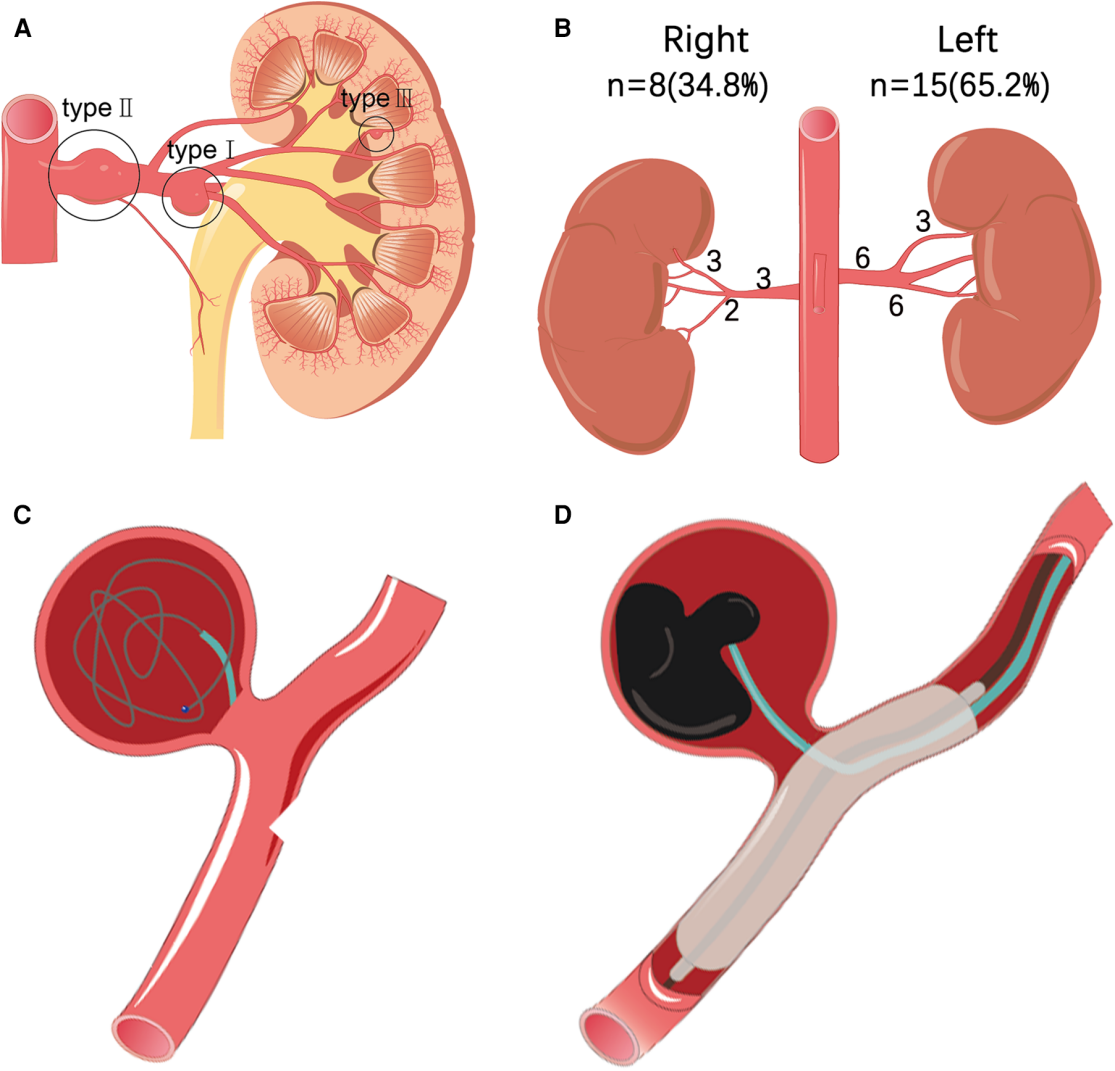
(**A**) According to the morphological classification of renal aneurysms: type I is located in the main or first-level branch of the renal artery saccular aneurysm, type II is a fusiform renal aneurysm, and type III is an interlobular renal aneurysm. (**B**) Statistics of the location and distribution type of 23 cases of renal aneurysms. (**C**) Interventional treatment plan for renal aneurysms, such as schematic diagram of stent coil embolization. (**D**) Interventional treatment plan for renal aneurysms includes a schematic diagram of liquid glue embolization.

Under local anesthesia, guided by a DSA (Siemens AritisZeego, Germany), the Seldinger technique was used to puncture the right or left femoral artery and insert a 5F Cobra catheter (Cook, USA) for abdominal aortic angiography and bilateral renal arteriography. To determine the size, location, size of aneurysm neck, number of renal aneurysms, and their relationship with parent artery, whether branched blood vessels, vascular malformations, vascular stenosis, and whether they are consistent with preoperative computed tomography angiography (CTA). Combined with DSA results, renal aneurysms were classified according to Rundback et al. (5) (Figure 1A): type I, successive aneurysm of the main renal artery or large segmental branch, type II, fusiform aneurysm, and type III, intralobar artery aneurysm. The treatment of RAA includes the following five types:

(1) Stent implantation: A guiding catheter is introduced. After the renal aneurysm was confirmed by angiography, a microguide wire was introduced into the distal end of the renal artery branch through the main trunk, and a self-expanding stent system (COOK, USA) was introduced to completely cover the neck of the aneurysm. The stent was then released, and an angiographic review was performed. After observing the condition of the aneurysm, a balloon was used to fully expand the stent when necessary.
(2) Coil embolization: The 5F Cobra catheter was inserted into the neck of the aneurysm, and coils (EV3, USA) were introduced into the aneurysm for embolization until the aneurysm was densely packed and there was no obvious flowing blood flow in the aneurysm lumen. Angiography confirmed that the parent artery was unobstructed, the aneurysm lumen disappeared, and the distal branch of the renal artery was not lost, indicating a successful operation (Figure 1C).
(3) Parent artery embolization: After selecting the cannula of the parent artery and angiography to further clarify the relationship between the renal aneurysm and the parent artery, a microcatheter and microguide wire were introduced to superselect the neck of the aneurysm. Simple coil embolization or liquid embolic agents N-butyl-2-cyanoacrylate (NBCA) (COMPONT,CHN) or Onyx (MEDTRONIC, USA）combined with coil embolization can be performed according to the inner diameter of the parent artery. The aneurysm and the parent artery were then observed by reexamination with angiography.
(4) Stent-assisted coiling embolization: A guiding catheter is introduced. After the renal aneurysm was confirmed by angiography, the microcatheter was introduced into the aneurysm lumen, the micro-guidewire was passed through the aneurysm body to the distal end of the renal artery branch, and the bare stent was introduced along the guidewire and positioned to the opening of the aneurysm. The coil was then inserted into the aneurysm body through a microcatheter to embolize the aneurysm.
(5) Liquid embolic agent embolization: The use of liquid embolic agents by experienced operators is a safe and effective method. As with other embolic materials, embolization also requires embolization of the distal and proximal ends of the parent artery. To reduce collateral ischemic events, it was recommended to use a balloon to temporarily seal the aneurysm neck (Figure 1D). Use antiplatelet drugs according to the patient's condition after operation. In the first, third and sixth months after operation, renal ultrasound, CT, CTA or MRA were selected to evaluate whether the aneurysm needs reperfusion, whether the stent has thrombosis or stenosis, and then imaging monitoring was performed once a year.

## Results

### Clinical outcomes

In 18 patients with RAA (age, 32–72 years, average age, 52.2 ± 11.2 years), a total of 23 aneurysms were found (aneurysm diameter 0.5–5.5 cm, average diameter 2.2 ± 1.4 cm) in which 11 (61.1%) were asymptomatic, which was accidentally discovered. The remaining patients had the following major complaints: four cases (22.2%) were diagnosed with low back pain, and one case (5.5%) showed low back pain with gross hematuria. The most common complication was hypertension, with nine cases (50%). Dyslipidemia was observed in four cases (22.2%). Three patients (16.7%) had a history of tobacco use. One patient (5.5%) had a splenic aneurysm. One patient (5.5%) had bilateral duplication of renal malformations and left adrenal pheochromocytoma. There were 15 aneurysms on the left side (63.6%) and eight on the right side (36.4%), of which one was bilateral with three aneurysms, three were unilateral with two aneurysms, and the rest were unilateral with single aneurysms (Table 1). Among the left aneurysms, six were type I, six were type II, three were type III, and of the right aneurysms: three were type I, three were type II, and two were type III (Figure 1B). One of the 23 aneurysms (4.3%) ruptured. Imaging revealed that four aneurysms had calcification, and two aneurysms with a diameter of <2 cm formed a mural thrombus.

**Table 1:** Clinical parameters of 18 patients with true renal aneurysm.

No.	Sex/Age (year)	Clinical Presentation	Comorbidities or Past Diseases	Aneurysm Size(cm)	Aneurysm Location
1	F/47	Flank pain	HTG	1.4 (L)	SIA
2	M/55	Incidental finding	CAHD, HTN, Tobacco use	1.4 (L)	SIA
3	F/40	Incidental finding	–	2.2 (R) 2.3 (R)	SIA SIA
4	F/48	Incidental finding	HTN, History of ICH	3.4 (R)	SIA
5	M/62	Flank pain	HTN	1.0 (R)	IRA
6	F/64	Incidental finding	HTN, SAA	5.5 (L)	IRA
7	M/32	Hypertension	HTG, Severe fatty liver	0.9 (R)	TMRA
8	F/48	Incidental finding	HTN, Left renal calculi	3.8 (L) 2.5 (L)	TMRA SIA
9	F/53	Incidental finding	Surgery history of breast cancer	4.0 (L)	TMRA
10	M/41	Flank pain	Tobacco use	1.4 (L)	TMRA
11	F/56	Incidental finding	HTN, Surgery history of thyroid adenoma surgery	2.6 (R)	TMRA
12	M/42	Hypertension	HTG, Tobacco use	2.5 (L) 1.8 (L) 0.7 (R)	TMRA TMRA TMRA
13	M/63	Incidental finding	HTN, DM, Duplex kidney, Left pheochromocytoma	1.3 (L)	SIA
14	F/46	Incidental finding	Bilateral kidney calculi	2.0 (L) 0.5 (L)	TMRA TMRA
15	M/76	Incidental finding	History of subtotal gastrectomy	2.0 (L)	TMRA
16	F/58	Incidental finding	Left renal calculi	1.5 (R)	SIA
17	F/66	Flank pain	HTN, DM, HCT, HTG, Surgery history of ICH	5.2 (L)	IRA
18	M/43	Flank pain + Gross hematuria	HTN	0.8 (L)	TMRA

Note: F, female; M, male; HTG, hypertriglyceridemia; HTN, hypertension; CAHD, coronary atherosclerotic heart disease; ICH, intracerebral hemorrhage; SAA, splenic artery aneurysm; RC, renal calculi; DM, diabetes mellitus; HCT, hypercholesterolemia; TMRA, the main renal artery; SIA, segmental + interlobar artery; IRA, intrarenal artery.

Five patients had not undergone endovascular interventional treatment for the following reasons. Case 5 was a 62-year-old male with lower back pain. DSA suggested an aneurysm in the right renal parenchyma with a diameter of approximately 1 cm. Since the aneurysm was not large in diameter, embolization may have caused a partial loss of renal function. Embolization was not performed after consultation with the patient. After 56 months, the aneurysm had not ruptured or enlarged. Case 7 was a 32-year-old male, an aneurysm was found due to increased blood pressure. The DSA showed an aneurysm (diameter, 0.9 cm) at the distal end of the right renal artery main trunk, accompanied by localized stenosis of approximately 90% of the right upper pole accessory renal artery. An increase in blood pressure is considered due to stenosis of the accessory renal artery. The aneurysm was not large in diameter; therefore, no intervention was performed. Case 8 was a 48-year-old female with left renal artery main and inferior posterior branch aneurysms, which were accidentally discovered during CT. The diameters were 3.8 and 2.5 cm, respectively. DSA showed difficulty in endovascular treatment; therefore, a surgical intervention was recommended. The follow-up patient underwent a kidney transplant at the outer hospital. Case 11 was an asymptomatic 56-year-old female; DSA revealed an aneurysm at the end of the right renal artery (diameter, 2.6 cm), involving multiple graded arteries. Therefore, surgical intervention was recommended. Case 12 was a 42-year-old male with elevated blood pressure. CT showed three bilateral aneurysms, of which two were on the left (diameters 2.5 cm and 1.8 cm, respectively). As multiple branches were involved, surgical intervention was recommended. Case 18 was a 46-year-old female with two unilateral aneurysms, which were accidentally found, of which the small size was approximately 0.5 cm and was not treated.

The remaining 13 patients with 14 aneurysms (aneurysms diameter, 0.7–5.5 cm; average diameter, 2.2 ± 1.5 cm) were treated endovascularly. Three aneurysms were treated with simple coil embolization, two with coils plus liquid embolic agent to embolize the aneurysm lumen, four underwent embolization of the parent artery embolization, one implanted with a bare stent, and two implanted ball-expanded stents, two used stent-assisted coil embolization. The technical success rate of the 13 patients with renal aneurysms was 100% and four (30.7%) had minor complications (mild abdominal pain, backache) after treatment. No serious complications or deaths occurred during treatment. After treatment, symptoms of low back pain were relieved in four patients, and blood pressure decreased in two cases. Four out of 18 patients were lost to follow-up, and the remaining patients were followed up for 4–89 months. There was no recurrence of the aneurysm or displacement of the stent or coil. Further details are provided in Table 2.

**Table 2:** 23 aneurysms treatment parameters in 18 patients.

No.	Aneurysm Size (cm)	Aneurysm Location	Aneurysm Type	Techniques	Materials	Results/ Compli -cations	Follow-up Imaging, Duration, and Outcome
1	1.4 (L)	SIA	I	CE + LEAE	NBCA and coil (QC-7-20-3D)	S/Abdominal pain	CT, 89 months, occluded
2	1.4 (L)	SIA	I	CE	Coil (QC-9-30-3D)	S/-	CT, 80 months, occluded
3	2.2 (R) 2.3 (R)	SIA SIA	III III	PAE + CE PAE + CE	NBCA and coil (QC-10-30-3D) NBCA and coil (QC-10-30-3D)	S/Lumbar soreness	CT, 4 months, occluded
4	3.4 (R)	SIA	II	CE + LEAE	Coil (QC-10-30-3D) and Onyx-18	S/-	CT, 12 months, occluded
5	1.0 (R)	IRA	III	–	–	–	CT, 56 months, stable size
6	5.5 (L)	IRA	III	PAE	Coil (QC-7-20-3D)	S/-	CT, 6 months, occluded
7	0.9 (R)	TMRA	II	–	–	–	CT, 30 months, stable size
8	3.8 (L) 2.5 (L)	TMRA SIA	II II	– –	– –	– –	CT, 32 months, Renal graft aneurysm
9	4.0 (L)	TMRA	I	–	–	–	Lost to follow-up
10	1.4 (L)	TMRA	II	SI	Self-expanding stent (ZIV5-18-125-6-20)	S/-	CT, 12 months, occluded
11	2.6 (R)	TMRA	I	–	–	–	Lost to follow-up
12	0.7 (R) 1.8 (L) 2.5 (L)	TMRA TMRA TMRA	II II II	SI – –	Self-expanding stent (ZIV5-18-125-6-30) – –	S/- – –	CT, 26 months, LK: stable size; RK: occluded
13	1.3 (L)	SIA	I	SACE	Self-expanding stent (ZIV5-18-125-6-20) and coil (QC-10-30-3D)	S/-	Lost to follow-up
14	2.0 (L) 0.5 (L)	TMRA TMRA	I III	CE –	Coil (QC-9-30-3D) –	S/Lumbar soreness –	CT, 20 months, occluded
15	2.0 (L)	TMRA	I	SACE	Self-expanding stent (ZIV5-18-125-6-20) and coil (QC-10-30-3D)	S/-	CT, 10 months, occluded
16	1.5 (R)	SIA	I	CE	Coil (QC-9-30-3D)	S/-	CT, 11 months, occluded
17	5.2 (L)	IRA	III	PAE	NBCA	S/-	MR imaging, 14 months, occluded
18	0.8 (L)	TMRA	II	SI	Self-expanding stent (ZIV5-18-125-6-20)	S/Lumbar soreness	CT, 13 months, occluded

Note: TMRA, the main renal artery; SIA, segmental + interlobar artery; IRA, intrarenal artery; CE, coil embolization; LEAE, liquid embolic agent embolization; PAE, parent artery embolization; SI, stent implantation; SACE, stent assisted coil embolization; S, success.

### Clinical features and treatment examples of typical renal aneurysms

**Figure 2:**
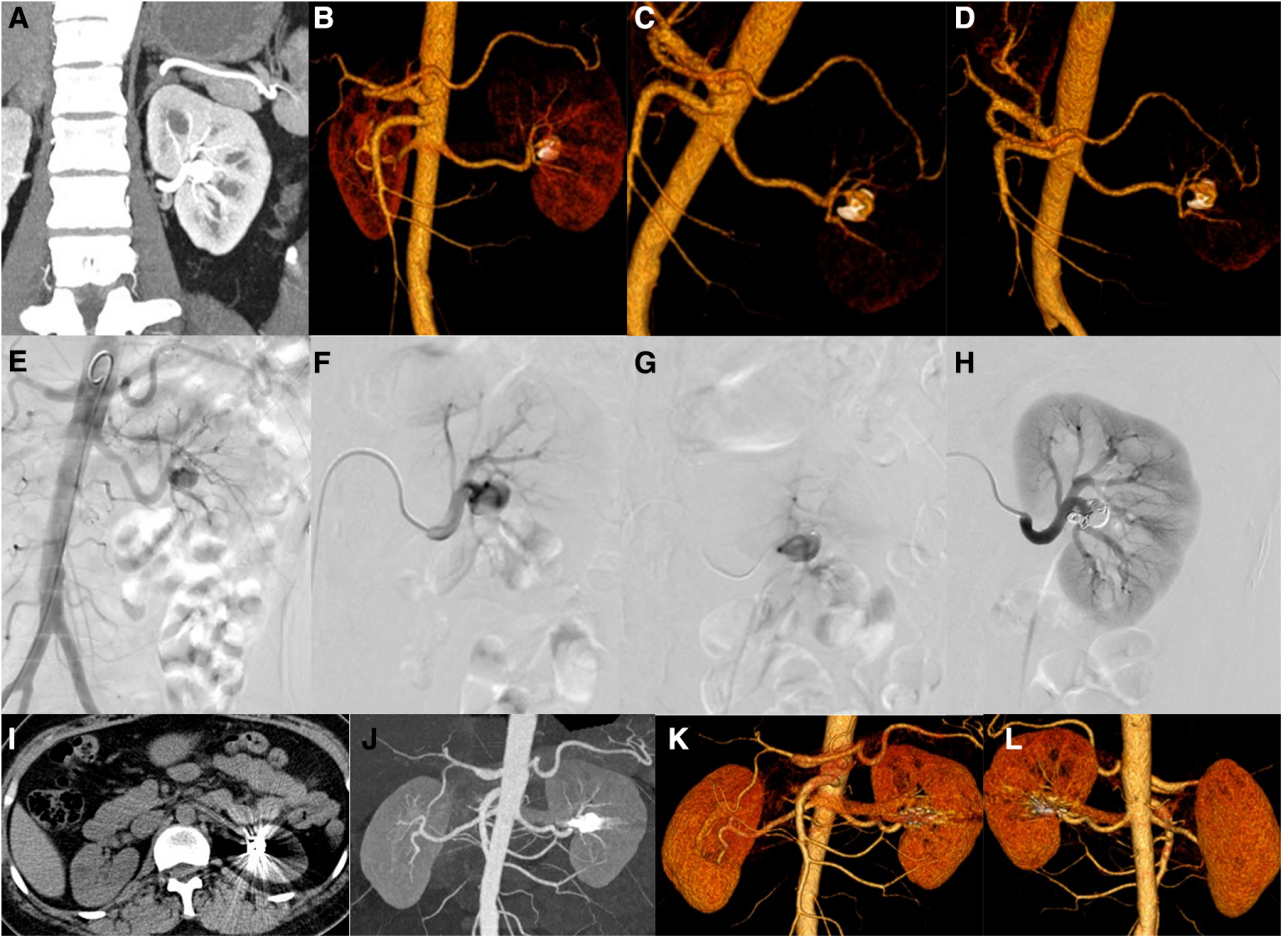
Patients no. 1 with renal aneurysms: (**A–D**) Computed tomography (CT) showed an aneurysm (1.4 cm × 1.2 cm) at the proximal end of the posterior segment of the left renal artery, accompanied by mural thrombus and peritumoral calcification, and several cysts in the right kidney and liver. (**E,F**) An angiography of the left renal artery is performed, and frontal imaging (digital subtraction angiography) showed that the left renal artery was equivalent to the artery adjacent to the left renal pelvis and showed a tumor-like expansion (approximately 1.1 × 1.4 cm). The left renal artery aneurysm is embolized with a microcoil and medical glue through a microcatheter, and the left renal artery angiography is repeated. (**I–L**) Three months after the operation, the CT scan showed that the posterior segment of the left renal artery showed a metallic fixation, which showed post-embolization changes.

Case 1 was a 47-year-old female with a major complaint of “repeated left back pain” for 1 month. Before the operation, CT showed an aneurysm of the proximal left renal artery posterior segment, approximately 1.4 cm in diameter (Figures 2A–D). DSA showed that the left renal artery was equivalent to the visible artery adjacent to the left renal pelvis, showing a tumor-like expansion, approximately 1.1 × 1.4 cm (Figures 2E–G). The left RAA was embolized using a coil and NBCA through a microcatheter. Angiography of the left renal artery showed that the aneurysm was embolized, and the artery was unobstructed (Figure 2H). Three months after the operation, the CT showed the posterior segment of the left renal artery with a metal-fixed shadow (Figures 2I–L).

**Figure 3:**
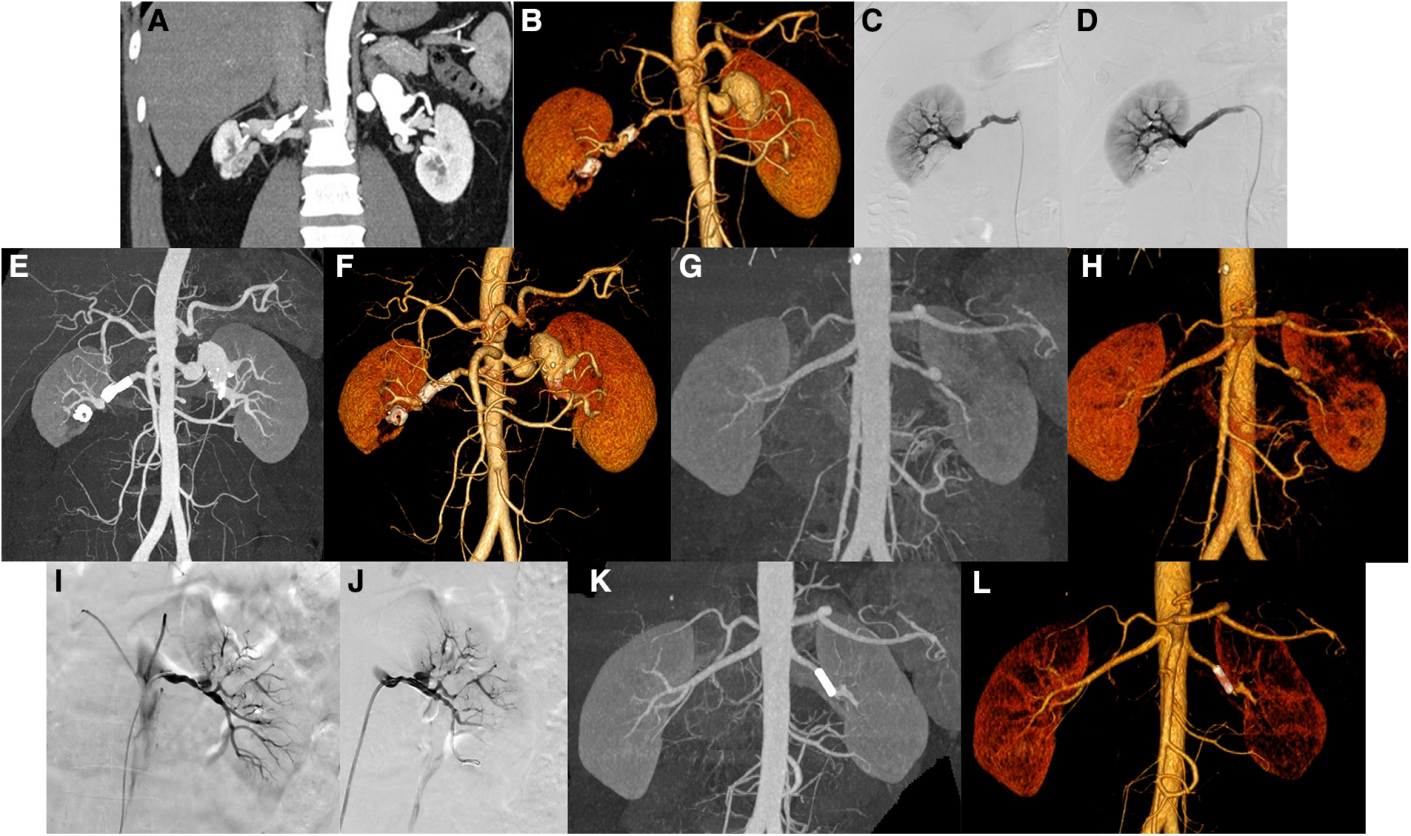
Patients no. 12 with renal aneurysms: (**A,B**) Computed tomography (CT) showed the main aneurysms of both renal arteries, with partial mural thrombosis and partial wall calcification, of which the main left renal artery is obvious (the widest part is approximately 2.5 cm in diameter). There are low-density foci in the lower right kidney near the renal sinus, with circular calcification at the edge, and adjacent right renal parenchyma with atrophy and ischemia. (**C,D**) The left and right renal arteries were performed, and frontal imaging (digital subtraction angiography) showed that the middle and distal parts of the left renal artery are enlarged (approximately 2.1 cm in diameter), involving intrarenal branches. There is approximately 60%–70% stenosis in the middle and distal part of the right renal artery, and the stenosis improved after stent implantation. (**E,F**) Three months later, CT showed that the stent lumen of the right renal artery is unobstructed. Patients no. 10 with renal aneurysm: (**G,H**) CT showed local dissection or formation of an aneurysm in the main trunk of the left renal artery and multiple ischemic abnormal enhancement areas in the left kidney. (**I,J**) Left renal artery angiography showed that the distal part of the left renal artery is approximately 80%–90% stenosis and a mung bean-sized aneurysm was observed. There is also 70%–90% stenosis in multiple branches of the left kidney. After stent implantation, the stenosis of the left renal artery is improved and covered the neck of the aneurysm. (**K,L**) CT scan 1 year after the operation showed that the left renal artery stent is in place, and the lumen is unobstructed.

Case 12 was a 42-year-old male who presented elevated blood pressure. Preoperative CT indicated two tumor-like expansions in the main left renal artery, the larger one involved the proximal segment of each branch, and the widest part was approximately 2.5 cm. And CT also indicated limited expansion of the main right renal artery approximately 0.6 cm wide (Figures 3A,B). DSA showed tumor-like expansion in the middle and distal parts of the left renal artery, which was obvious in the distal part, with a diameter of approximately 2.1 cm, involving the intrarenal branches; the middle and distal parts of the right renal artery had a stenosis of approximately 70%, while the distal part expanded with a diameter of approximately 7.3 mm (Figure 3C). The right renal artery angiography was performed again, the stenosis improved after stent implantation, and the tumor-like expansion disappeared (Figure 3D). The follow-up CT showed that the lumen of the stent was unobstructed, and the size of the main left RAA was the same as before (Figures 3E,F).

Case 10 was a 41-year-old male with a sore left waist for half a month. Preoperative CT showed that the central part of the left renal artery was enlarged in a tumor-like shape, with a size of approximately 1.0 × 1.4 cm, with a vaguely visible intima. The proximal and distal blood vessels of the left renal artery were irregularly narrowed; the narrowest part was approximately 80% (Figures 3G,H). It is considered a local dissection or aneurysm formation in the main trunk of the left renal artery. Renal dynamic imaging before surgery showed left glomerular filtration rate (LGFR) of 28.11 ml/min and right glomerular filtration rate (RGFR) of 48.29 ml/min. DSA showed the distal end of the left renal artery was stenosis (approximately 80%–90%), and a mung bean-sized aneurysm was observed in the left kidney. A stent was implanted in the distal part of the main artery to cover the neck of the aneurysm (Figures 3I,J). Angiography was performed again, and the stenosis of the left renal artery trunk improved after stent implantation, and no aneurysm was observed. One year postoperatively, a CT reexamination showed that the stent was in place, the visualization of the cavity was unobstructed, and there was no internal leakage or extravasation of the contrast agent (Figures 3K,L). Reexamination of renal artery imaging LGFR 35.77 ml/min, RGFR 50.81 ml/min, and the glomerular filtration rate improved compared to previous.

**Figure 4:**
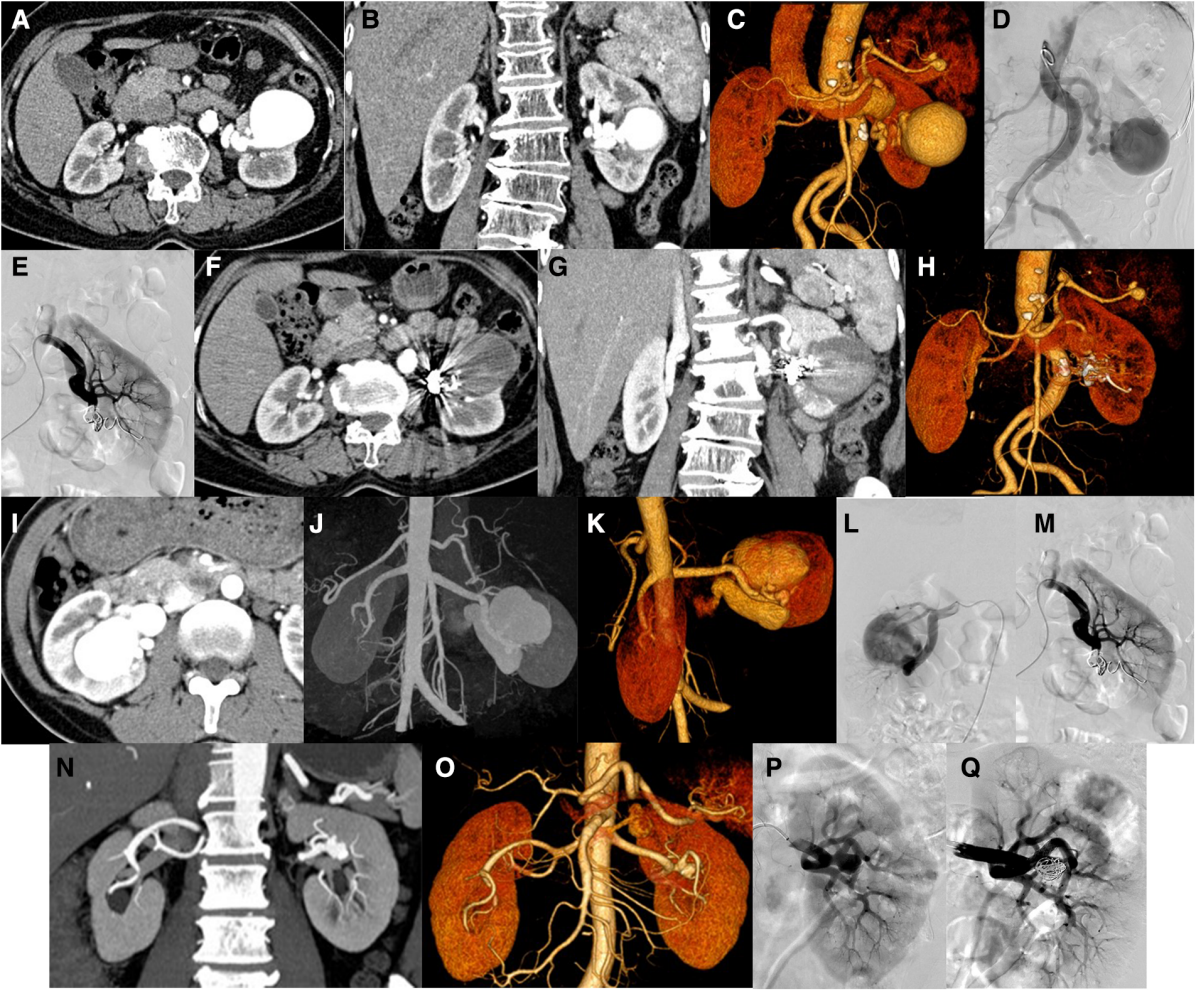
Patients no. 6 with renal aneurysm: (**A,B**) Computed tomography (CT) showed that the distal branch of the left renal artery (equivalent to the middle and lower part of the left kidney) showed a cystic dilation (approximately 4.9 cm × 4.5 cm × 4.9 cm), and multiple tumor-like dilations occurred in the proximal part of the splenic artery and the lumen near the splenic hilum. (**C,D**) An angiogram of the left renal artery showed that the left renal artery thickens, equivalent to a saccular dilated blood vessel (approximately 5.5 cm in diameter) in the lower-left kidney. Left renal aneurysm was considered. After embolization of the blood supply artery with a controllable coil, the above-mentioned saccular dilated blood vessels were not observed after repeated angiography. (**F–H**) Reexamination of the CT scan 1 week after the operation showed that the original distal branch (equivalent to the middle and lower part of the left kidney) showed cystic dilation, but no enhancement was observed. Patients no. 3 with renal aneurysm: (**I–K**) Enhanced CT showed enlargement of the right kidney and lesions in the area of the right kidney. Considering that the arteriovenous malformation may be accompanied by aneurysm-like expansion (maximum diameter approximately 4.7 cm), an arteriovenous fistula. (**L,M**) An angiogram of the right renal artery showed two large aneurysms in the middle and lower Poles of the right renal artery, whose sizes were 2.2 cm × 1.8 cm and 2.3 cm × 1.0 cm and they were in series. The responsible blood vessel of the right renal artery was embolized with microspring through the microcatheter, and Glubran was used for embolization. After embolization, the right renal aneurysm disappeared, and the right renal parenchyma was well displayed. Patients no. 13 with renal aneurysm: (**N,O**) Left renal aneurysm (maximum inner diameter of approximately 12.7 mm), and the left adrenal gland with a rich blood supply occupying the space, considering the possibility of pheochromocytoma or adenoma, bilateral repeated renal malformations. (**P,Q**) An angiogram of the left renal artery showed a wide-base aneurysm (approximately 11 mm in diameter) in the branch of the left renal artery and were placed at the base of the parent aneurysm with a stent. After embolization with a controllable coil, most of the aneurysm is blocked, the abdominal aorta is tortuous.

Case 6 was a 64-year-old female with an abnormal vascular cluster in the left renal parenchyma that was accidentally detected during CT examination. The size is approximately 4.9 cm × 4.5 cm × 4.9 cm. Considering vascular malformations, arteriovenous malformation (AVM) is more likely (Figures 4A–C). DSA showed the left renal artery thickened on angiogram, equivalent to the saccular dilated blood vessel in the lower part of the left kidney, with a diameter of approximately 5.5 cm. In this case, aneurysms were also considered. The blood supply artery was embolized with a controllable coil, and reimaging did not show the saccular dilated blood vessels mentioned above (Figures 4D,E). The distal branch of the left renal artery showed a cyst-like expansion with a size of approximately 4.9 cm × 4.5 cm × 4.9 cm, and no enhancement was observed (Figures 4F–H).

Case 3 was a 40-year-old female with a mass in the right kidney on physical examination. Preoperative CT examination revealed that the right kidney was enlarged, showing masses and earthworm-shaped blood vessel masses (Figures 4I–K). DSA showed two giant aneurysms were observed in the middle and lower poles of the right renal artery (Figure 4L), with diameters of 2.2 and 2.3 cm, respectively, in a tandem state, showing multivessel supply. The right renal artery was embolized with a coil through a microcatheter and embolized with NBCA. After embolization, the right aneurysm disappeared on another image (Figure 4M).

Case 13 was a 63-year-old man. A left renal aneurysm was found during CT examination due to a left pheochromocytoma. The maximum inner diameter was approximately 12.7 mm, the opening was approximately 2.5 mm wide, and the vessel wall showed shell-like calcification (Figures 4N,O). DSA showed an aneurysm with a diameter of approximately 1.1 cm with a wide base in branch of the left renal artery. Stents were placed at the base of the parent artery, and the aneurysm was embolized with a coil. Another angiogram revealed that the aneurysm was blocked (Figures 4P,Q).

## Discussion

Renal aneurysms (RAAs) are rare clinical entities, and there are few reports on their natural course. Henriksson et al. (6) reported a total of 34 RAAs in 21 patients with an average follow-up of 102 months (55–204 months), and none of them ruptured. Tham et al. (7) reported that 69 patients with RAA were treated conservatively and followed up for an average of 4.3 years without aneurysm rupture. McCarron et al. (8) reported that 19,600 autopsies performed at New York Hospital did not reveal a ruptured renal aneurysm. These studies indicate that RAA has a benign natural course. Renal aneurysms usually grow slowly, and some studies report that the median annual growth rate of true renal aneurysms is 0.06–0.6 mm (9–11). RAA is insidious with no obvious clinical manifestations. Most of these were accidentally discovered. A small number of patients have symptoms, such as hypertension, hematuria, and low back pain. Potential complications of RAA include thrombosis, renal infarction, and rupture. Rupture and hemorrhage are the most serious complications, and preventive interventions are required in some cases.

RAA can be caused by many factors, such as congenital disease, dilatation after stenosis, arteritis, and infection, among which the most common is atherosclerosis and fibromuscular dysplasia (FMD) (12, 13). Henke et al. found that arterial FMD is the most common vascular disease associated with RAA (14). FMD of the renal arteries is associated with renal aneurysms in females (15). Females represent 72% of patients with RAA (3). Therefore, the American Society of Vascular Surgery recommends screening for FMD in females with RAA (16).

Aneurysms can be diagnosed and evaluated using various imaging methods, each with its advantages. DSA is an invasive test, but it can not only determine the location, size, and number of aneurysms, but also provide opportunities for interventional therapy. It is also the gold standard for diagnosing renal aneurysms (PMID: 24363127). CTA is the first choice for diagnosing RAA. It displays the main renal artery and branches of grades 1–3 in a three-dimensional manner and obtains all information on the renal artery and renal vein (16). However, it is difficult to completely observe RAA with a smaller diameter in the conventional cross-section of CTA, and it is easy to miss the diagnosis. The diagnosis of RAA by magnetic resonance angiography (MRA) is based on the absence of radiation or contrast medium nephrotoxicity; therefore, it is especially suitable for pregnant women. When RAA is complicated by renal artery stenosis, MRA overestimates the degree of stenosis due to signal loss and the examination is relatively expensive. Doppler ultrasound is a cheap and noninvasive technique for detecting renal aneurysms. However, this technique is highly dependent on the position of the patient and the operator's ability, which may cause interpretation defects or limited visibility during the inspection process. Furthermore, RAAs can be misdiagnosed as cysts or renal vein dilation during ultrasound examinations (17). A multicenter study found that CTA (58%) was the most commonly used method for the diagnosis and evaluation of RAA, followed by non-contrast-enhanced CT (24%), MRA (6%), catheter angiography (5%), and ultrasound (4%) (10).

Pregnancy is believed to be associated with renal aneurysm rupture and mortality. The important reasons for the increase and rupture of aneurysms during pregnancy are mainly due to increased blood volume, increased intra-abdominal pressure, changes in hormone metabolism, and hyperdynamic status. Most studies have shown that most RAA ruptures occur in the third trimester (18–20). Previous studies have reported a mortality rate of renal aneurysm rupture during pregnancy as high as 56%–92% for pregnant women and 82%–100% for fetuses (19). Modern studies report the mortality of renal aneurysm rupture during pregnancy, among which the maternal mortality rate was 34%–58% and the fetal mortality rate was 60%–78% (3, 20). The mortality rates of the two have decreased significantly, which is related to the progress of interventional medicine.

Solitary kidneys with RAAs are clinically rare. As patients with isolated kidneys have poor renal compensatory ability, once pathological changes occur, renal function should be protected as much as possible during treatment to avoid renal parenchymal damage. In all surgical procedures, up to 5% of cases have undergone unplanned nephrectomy; therefore, endovascular techniques should be considered the preferred technique for isolated cases of renal aneurysm (21).

The main purpose of RAA treatment is to prevent rupture of renal aneurysms, relieve secondary hypertension, and protect kidney function. However, the indications for RAA treatment remain controversial. Most surgeons generally believe that if the RAA is not ruptured, preventive surgical intervention is required if the following conditions exist: (1) large size (>2 cm), (2) symptoms, (3) refractory hypertension with significant renal artery stenosis or thromboembolism, (4) childbearing age (for women) (3, 4, 22). However, Lawrence et al. (9) stated that surgery to prevent rupture of asymptomatic RAA with a defined >2 cm might be too aggressive because asymptomatic RAA rarely rupture and grow slowly. In the 2020 American Society of Vascular Surgery Guidelines for the Diagnosis and Treatment of Visceral Aneurysms, it is recommended that the maximum diameter of the aneurysm should be 3 cm as the critical value for patients with renal aneurysms without complications. However, the article also noted that branch aneurysms or saccular aneurysms have a high risk of rupture; therefore, invasive treatment may be required regardless of their size (16). Some studies also indicate that for pregnant women or women of childbearing age, the maximum diameter of the aneurysm of >1.5 cm is considered a clear indication for surgery (23). However, multiple guidelines recommend that women of childbearing age with renal aneurysms undergo surgical treatment regardless of aneurysm diameter (16, 23, 24).

Surgical treatment of renal aneurysms mainly includes traditional open surgery, laparoscopic surgery, and endovascular treatment. The specific surgical method is selected based on the aneurysm anatomy, location, origin artery, and conditions of the patient. The specific surgical method is selected based on the aneurysm anatomy, location, origin artery, and conditions of the patient. A previous study did not show significant differences in mortality, complication rate, reintervention requirements, and length of stay after endovascular treatment compared with open surgery, but the incidence of cardiovascular and cerebrovascular events was higher in open surgery (25). Furthermore, the endovascular treatment group had shorter operation times, lower blood loss, and shorter hospital stay in the intensive care unit (26, 27). Studies have shown that the success rate of endovascular treatment is 73%–100%, and the perioperative complication rate is 13%–60% (3, 28–30).

Endovascular treatment techniques for RAA include stent placement, selective coil embolization, parent artery embolization, stent-assisted coil embolization, balloon-assisted coil embolization, and selective fluid embolization. Among them, coil embolization is the most extensive embolization technique used to treat the three types of aneurysms. Type I aneurysm: Stent placement is widely used in Type I renal aneurysms, whereas its application in Type II branch renal aneurysms is more limited (may cover important branches) (31). The stent was placed in an aneurysm 15 mm from the orifice of the renal artery to provide a sufficient landing area for the stent, and the aneurysm sac had no related branches. Stent transplantation can be used to treat RAA combined with renal artery stenosis. However, the stent graft is difficult to bend and may cover important branches of the renal artery and cause renal infarction. However, the stent graft is difficult to bend and may cover important branches of the renal artery and cause renal infarction. Blood clots can form in small arteries after stent placement. Therefore, stent grafts are not recommended for renal aneurysms with small blood vessels (<6 mm) or curved vessels (32). Selective coil embolization is also commonly used for saccular aneurysms with a neck diameter of <4 mm or an aneurysm-to-neck ratio of 2:1 (33). Type II aneurysm: Due to the special anatomy and location of Type II aneurysms, intravascular interventional therapy is challenging. Open vascular surgery, such as external reconstruction, autotransplantation, and nephrectomy, is the standard treatment for fusiform aneurysms in the main renal artery or large segment artery, such as external reconstruction, autotransplantation, and nephrectomy (22, 33). Some studies reported that multilayer stents have been used successfully to treat complex renal aneurysms (34). Multilayer blood flow regulators can promote the conversion of turbulent flow to laminar flow, induce organizing thrombosis in the aneurysm lumen, reduce occlusion of bypass branch vessels, and broaden the application of stents in complex renal aneurysms (35). Type III aneurysm: Superselective embolization of the parent artery was achieved using liquid embolic agents and coil embolization. It is used to treat aneurysms with less kidney tissue or a larger aneurysm lumen from a parent artery. The small arteries originating from the aneurysm are occluded, leading to an infarction of the corresponding renal parenchyma, which is mostly clinically asymptomatic, or symptoms are adequately treated.

For patients with renal aneurysms undergoing treatment, it is recommended to complete a CTA, MRA, or DSA imaging examination before discharge (16, 33). They will be followed up and evaluated at 1 month, 6 months, and every year (16, 36). For patients undergoing nonsurgical treatment, it is recommended to conduct a review every year until two consecutive studies are stable, after which the review time can be extended to once every 2–3 years (16).

In summary, endovascular interventional treatment of renal aneurysms has the advantages of a high technical success rate, fewer complications, less trauma, a shorter hospital stay, and a lower reintervention rate. Its safety and efficacy are worth recognizing, and it has become the first choice of treatment for renal aneurysms. As technology matures, an increasing number of complex renal aneurysms can undergo endovascular interventional therapy. Long-term follow-up is warranted to rule out the possibility of aneurysm reperfusion.

## Data Availability

The original contributions presented in the study are included in the article/Supplementary Material, further inquiries can be directed to the corresponding author/s.
